# Self-directed behaviors differentially explain associations between emotion dysregulation and eating disorder psychopathology in patients with or without objective binge-eating

**DOI:** 10.1186/s40337-020-00294-4

**Published:** 2020-05-01

**Authors:** Elin Monell, David Clinton, Andreas Birgegård

**Affiliations:** 1 Centre for Psychiatry Research, Department of Clinical Neuroscience, Karolinska Institute, and Stockholm Health Care Services, Stockholm County Council, Norra Stationsgatan 69, SE-11364 Stockholm, Sweden; 2 Department of Medical Epidemiology and Biostatistics, Karolinska Institutet, Stockholm, Sweden; 3 Institute for Eating Disorders, Oslo, Sweden

**Keywords:** Eating disorders, Emotion dysregulation, Self-directed behaviors, Mediation analysis, Objective binge-eating episodes, DERS

## Abstract

**Background:**

Emotion dysregulation and negative self-directed behaviors are key characteristics of eating disorders (EDs), but their interaction in relation to ED psychopathology is insufficiently explored, and empirically robust and clinically relevant models are needed.

**Methods:**

This study examined whether the association between emotion dysregulation and ED psychopathology was mediated by different negative self-directed behaviors in 999 ED patients divided into two sub-samples based on absence or presence of objective binge-eating episodes (OBE). Several simple and extended mediation models were examined using the Difficulties in Emotion Regulation Scale (DERS) as independent variable, the Structural Analysis of Social Behavior (SASB) as mediator, and the Eating Disorder Examination Questionnaire (EDE-Q) as dependent variable.

**Results:**

An associational pathway was found where higher emotion dysregulation was associated with more negative self-directed behaviors, which in turn was associated with higher ED psychopathology. Self-directed behaviors of importance differed between patient groups. In participants without OBE, lower self-love and higher self-attack were influential, whereas in participants with OBE, lower self-affirmation and higher self-blame were influential.

**Conclusions:**

Self-directed behaviors may help to explain the association between emotion dysregulation and ED psychopathology. Our findings have both theoretical and clinical implications that are pathology-specific. Addressing specific self-directed behaviors could be an important way of helping patients deal with their emotions in relation to ED psychopathology.

## Plain English summary

Eating disorders are complex serious psychiatric conditions but the understanding of how they develop and are maintained is unclear, while treatment outcomes are mixed. This study examined difficulties understanding and managing emotions (emotion dysregulation) and the habitual way an individual internally treats and regulates him−/herself (self-directed behaviors), in relation to eating disorder psychopathology among 999 patients with eating disorders. We found that higher emotion dysregulation, such as difficulties focusing on and understanding emotions, was associated with negatively attuned self-directed behaviors, for instance with harsh self-criticism, which in turn was associated with greater body, shape and weight concerns. We found that in participants who did not have objective binge-eating episodes, higher emotion dysregulation seemed to be associated with less self-love and more self-attack in relation to eating disorder symptoms. In participants who did have objective binge-eating, higher emotion dysregulation was associated with less self-affirmation and more self-blame. Our results suggest that negative types of self-directed behaviors may maintain problematic patterns of emotional difficulties and eating disorder symptoms and that this needs to be addressed in treatment.

## Introduction

Eating disorders (EDs) are complex psychiatric conditions associated with high rates of psychiatric and medical comorbidity, life-disruptions, and significant suffering [1], but the etiology of EDs is unclear [2] and treatment outcomes are mixed [3]. In order to improve our understanding and treatment of EDs we need to identify empirically robust and clinically relevant models of etiology and maintenance. Previous research suggests that EDs are characterized by emotion dysregulation and negative self-directed behaviors [4, 5]. Emotion dysregulation refers to difficulties understanding and managing emotions [6], while self-directed behaviors refers to dimensions of self-control vs. spontaneity and self-affiliation vs. self-attack [7]. Independently, these factors impact symptoms, but their interaction in relation to ED psychopathology remains insufficiently explored. Recently, Monell, Högdahl, Forsén Mantilla, and Birgegård [8] suggested that the effect of emotion dysregulation on ED psychopathology in female students was mediated through more negative self-directed behaviors. Increased emotion dysregulation (e.g., losing control over one’s behavior when in distress) was associated with negative self-directed behavior (e.g., harsh self-criticism), which in turn was associated with greater ED psychopathology. The present study aims to replicate and extend these findings in a large clinical ED sample, hoping to identify potential intervention targets that closely reflect patients’ experience of emotion dysregulation.

### Dimensions of emotion dysregulation and associations with ED pathology

Emotion dysregulation is associated with ED pathology [9, 10] and ED outcome [11]. Further, behavioral ED symptoms may represent dysfunctional emotion regulation strategies in response to negative affect [12]. However, ‘emotion dysregulation’ as a concept is imprecise as it has been given different meanings and has been measured using a variety of instruments. Converging on a common model, several recent ED studies have employed the multidimensional model of Gratz and Roemer and the Difficulties in Emotion Regulation Scale (DERS) [6]. This model was developed to capture the following four dimensions.

*Reduced emotional awareness and clarity* describes an inability (or unwillingness) to focus on emotional signals and an insufficient understanding of them, resembling alexithymia (i.e., difficulties identifying and describing emotions) [13]. Alexithymic traits are considered key characteristics of restrictive ED pathology [14]. However, research indicates its relevance across the entire ED diagnostic spectrum [15], and difficulties in emotional awareness and clarity may distinguish ED patients generally from controls [9]. *Non-acceptance of emotional distress* describes tendencies to respond with negative secondary emotions (e.g., self-directed anger, shame) towards one’s own distress. It has been argued that non-acceptance and avoidance of emotions is a key maintaining factor in anorexia nervosa (AN) [16]. Non-acceptance is associated with both overall ED psychopathology [9] and restraint [17]. *Difficulties maintaining impulse control and goal-directed behaviors when upset* refers to difficulties controlling, or fear of losing control, over one’s reactions and behavior when in distress. This resembles negative urgency (i.e., engaging in rash and impulsive behavior when distressed), particularly relevant for patients with binge-eating [5, 18]. *Perceived lack of emotion regulation strategies when upset* describes a sense of emotional helplessness and a tendency to surrender to negative emotions when upset. It has the strongest unique association with ED psychopathology in both mixed clinical EDs and controls [9, 19]; improvement in this dimension has been associated with better treatment outcome for binge-eating patients [20].

In summary, lower emotional awareness and clarity may differentiate patients from controls, difficulties maintaining impulse control and goal-directed behaviors when upset may characterize binge-eating pathology, and higher levels of perceived emotional helplessness are associated with higher ED psychopathology regardless of diagnostic status.

### Self-directed behaviors and associations with ED pathology

**Fig. 1:**
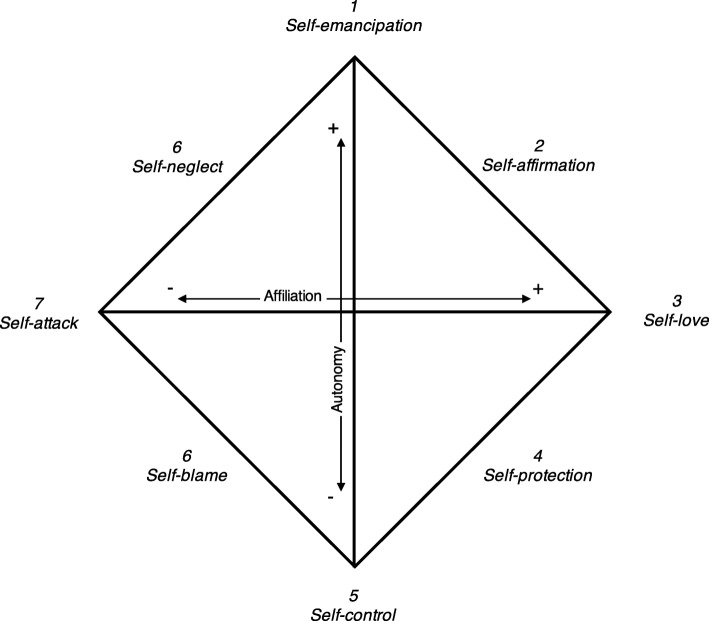
Structural Analysis of Social Behavior Intrex model Cluster version. From: Benjamin LS. Interpersonal Diagnosis and Treatment of Personality Disorders, 2nd ed. New York: The Guilford Press, 1996

Low self-esteem is characteristic of EDs [21], and patients’ sense of self-worth is often determined by body weight and shape [22]. However, while the sense of self is a complex phenomenon, most self-related concepts and measures capture unidimensional aspects of self-evaluation and self-directed feelings (i.e., approve-disapprove, like-dislike etc.). In interpersonal theory, the self is conceptualized in terms of the habitual way an individual internally relates to him−/herself [7], and thus it refers to self-directed behaviors. The Structural Analysis of Social Behavior (SASB) model and its accompanying measure [23] organizes self-directed behaviors in a circumplex (see Fig. 1). The horizontal *Affiliation* axis captures affective valence, while the vertical *Autonomy* axis captures self-regulation; combinations of these axes form different types of self-directed behaviors, grouped into the following eight clusters.

*Self-emancipation* describes spontaneous/free self-regulation as opposed to strict *self-control*. Control and compulsivity are central themes in EDs, particularly AN [24]. ED symptoms have been described as strategies to manage perceived lack of self-control [25], while actual loss of control (over eating) is central in binge-eating pathology. In AN, self-emancipation and spontaneity seems decreased [26, 27], while self-control is higher relative to other EDs [26]. Higher self-control in AN is associated with worse 3-year outcome [28] and increased 12-month suicidal ideation [29]. *Self-affirmation* describes a friendly, accepting, and curious stance toward the self. Self-affirmation, often strikingly limited in EDs, has received increased attention in novel treatments [30]. *Self-blame* instead describes harsh and hostile self-regulation. Self-blame strongly resembles self-criticism and maladaptive perfectionism, both common ED traits [21]. Low self-affirmation and high self-blame are associated with higher ED psychopathology in clinical, high-risk and normal samples [4]. *Self-love* and *self-attack* are most similar to self-esteem. Lower self-love is associated with higher ED psychopathology [4]. Higher initial self-hate predicts worse outcome in various EDs [28, 31], while higher initial self-love predicts better outcomes in all EDs [31]. *Self-protection* describes active engagement in activities perceived as beneficial for the self and protection of self-interests, while *self-neglect* describes negative autonomy, such as ignoring one’s own needs. Although self-protection can be considered a positive behavior, it predicts negative outcome in AN [28], possibly because some patients may perceive their symptoms as the best way of taking care of their needs. More intuitively, higher self-neglect predicts worse outcome in AN [28, 31].

In summary, lower self-affirmation and higher self-blame have strong concurrent associations with higher ED psychopathology in various samples, while lower self-love and higher self-attack predict outcome in several EDs. The associations with self-protection and self-control is distinctive of AN.

### Pathways whereby emotion dysregulation and self-directed behaviors may influence ED psychopathology

Several ED etiology and maintenance models include both emotion and self-related aspects [32]. These include for instance the transdiagnostic maintenance model of EDs [21], i.e., ‘mood intolerance’, ‘clinical perfectionism’, and ‘core low self-esteem’; the cognitive-interpersonal maintenance model of AN [33], i.e., alexithymia, avoidant emotion processing, and compulsivity; and the model underlying integrative cognitive-affective therapy for bulimia nervosa (BN) [34], i.e., emotion dysregulation, self-directed behaviors, and self-discrepancy. An additional model highlights shame, self-criticism, and lack of self-compassion [35].

Evidence for how various emotion dysregulation dimensions and specific self-directed behaviors may influence ED psychopathology is however lacking. Existing models use different terms, study isolated aspects of emotion regulation (e.g., alexithymia) and self-related cognition/behavior (e.g., self-esteem), and few examine a wide range of such constructs simultaneously. Due to different focus on risk versus maintenance factors, they are also difficult to compare and evaluate in terms of potential association chains; one factor being associated with another that in turn might be associated with symptom expression. Mediation models can help delineate such association pathways by which emotion dysregulation and self-directed behaviors could be associated with ED psychopathology. Since the DERS and SASB models encompass important concepts in several theoretical models, their combination may provide an opportunity to integrate existing models, disentangle association pathways, and increase model specificity.

To our knowledge, only our own previous study has concurrently used the DERS and the SASB in relation to ED pathology [8]. Here, we found that higher emotion dysregulation, mediated by negative self-directed behaviors, was associated with greater ED psychopathology in female students [8]. However, this study neither examined ED patients nor specified different emotion dysregulation dimensions or types of self-directed behaviors. Clinically, it may be useful to know if different emotion dysregulation dimensions are associated with ED psychopathology through distinctive types of self-directed behaviors in ED patients. For instance, perceptions such as “my emotions are shameful and I hate to have them” might be associated with “I’m useless and I need more discipline”. This, in turn, may fuel attitudes such as “I have to control my ugly body”. Empirically disentangling potential significant association chains between emotion dysregulation, self-directed behaviors, and ED psychopathology could help therapists approach clinical phenomena accurately while remaining close to patients’ subjective experiences.

### Aims

Given the diagnostic diversity of eating EDs it is important to study mediational pathways in groups of patients with or without particular core symptoms. However, diagnostic migration is common within EDs [36], so specific ED diagnoses may have low clinical and predictive validity [37]. Therefore, diagnosis-specific models may be overly narrow and of limited clincal utility. Even so, there appears to be some differences in emotion and self-related processes between primarily restricting patients and those with loss of control binge-eating, possibly reflecting underlying differences in impulsivity [38]. Such differences may entail differential risk for specific core symptoms; impulse control difficulties and negative urgency seemingly more associated with binge-eating [39–41], whereas over-controlled regulation and compulsivity instead seem more associated with pathological restrictive eating without binge-eating [24, 42]. The current study will therefore group EDs into broader categories depending on presence or absence of loss of control binge-eating (defined as objective binge-eating episodes; OBE) in order to capture a wider and more ecologically valid clinical picture.

We tested whether the association between emotion dysregulation and ED psychopathology is mediated by negative self-directed behaviors in ED patients with or without OBE, expecting to replicate the main finding by Monell et al. [8] in both groups. We also extend our previous work by exploring whether particular emotion dysregulation dimensions are mediated by distinct self-directed behaviors. Here, we had no specific hypotheses regarding which self-directed behaviors would be mediators in which model, or if there would be differences between groups.

## Method

### Participants

The sample was drawn from a clinical database covering specialized ED units in Sweden (the Stepwise database) [43]. Stepwise inclusion criteria are self- or medical referral to a treatment unit, an ED according to the Diagnostic and Statistical Manual of Mental Disorders 4th version (DSM-IV) [22], and intent to treat from the unit. Stepwise includes patients with the full range of DSM-IV EDs of all ages entering treatment since 2005. Stepwise initial assessment, performed by trained ED professionals by the patients 3rd visit to the unit, includes semi-structured interviews, clinical ratings and self-ratings (both mandatory and optional, all in Swedish), and takes around 45 min. DSM-IV ED diagnoses are based on the Structured Eating Disorder Interview (SEDI) [44] with good validity. The present sample consisted of 999 female patients aged 16–72 years (*M* = 24.8, *SD* = 8.4) with AN restrictive subtype (AN-R; *n* = 172), AN binge/purge subtype (AN-BP; *n* = 64), BN (*n* = 350), binge eating disorder (BED; *n* = 40), and other specified feeding and EDs (OSFED; *n* = 373).

Data were extracted on October 30th 2016; extraction procedure, exclusions, and rationale for re-categorization from DSM-IV into DSM 5th version (DSM-5) diagnoses [45] are reported in detail elsewhere [9]. The Eating Disorder Examination Questionnaire (EDE-Q) and the SASB are mandatory in the Stepwise assessment while the DERS was included in Stepwise in 2014 as an optional instrument at the individual patient level (i.e., clinicians decided whether to include DERS or not). DERS was administered to 37% of patients during this study time frame. Patient characteristics (e.g., ED pathology, anxiety, depression, age, ED-duration) did not seem to influence if they were administered the DERS or not; instead, clinician/clinic variables seemed more influential (for more details, see Monell et al.) [9]. Additionally, SASB variables did not differ between patients with or without DERS-ratings (using independent *t*-tests; *p*s > .01).

The sample was then split into two groups depending on presence/absence of OBE. Patients with AN-R were categorized into non-OBE EDs; patients with BN and BED into OBE EDs. For patients with AN-BP and OSFED, symptoms can include OBE (in AN-BP, the binge/purge may in some cases refer to purging only), and self-rated presence of OBE in the last month (using self-rated ED-pathology, see measures) decided their group. The study was approved by the Stockholm regional ethics committee (2015/928–31/4).

### Measures

*The DERS* [6] measures self-rated emotion dysregulation in 36 items scored 1–5, providing a Total score and six subscales: Non-Acceptance, Goals, Impulse, Awareness, Strategies, and Clarity. Items are summed to form subscales and the Total score is the sum of all scores; higher scores indicate more emotion dysregulation. The validity and psychometric properties of the subscales are debated [46], and item content might not always match intended theoretical concept [47]. Based on such criticism, rather than using the six subscales and in order to increase specificity, this study focused on item content in subscales corresponding to the original four dimensions in the extension analyses.

Subscales Awareness and Clarity were summed to form the first dimension (range 11–55). As shown using bifactor modelling, Awareness and Clarity capture high degrees of unique information beyond a general emotion dysregulation factor (48, using largely the same sample as this; 50) and have been combined in previous research [13, 48]. They also correlate strongly, primarily with each other in our sample (see Tables S1 and S2). Non-Acceptance alone formed the second dimension (range 6–30), and Goals and Impulse were summed to form the third (range 11–55). Goals and Impulse capture similar low degrees of unique information [46, 49] and correlate strongly with each other in our sample (Tables S1 and S2). Strategies, that captures the least unique information [46, 49], and thus corresponds to a general emotion dysregulation factor, formed the fourth dimension (range 8–40). The Total score (range 36–180) was used in replication analyses. Apart from the debate on the subscales, the DERS has shown good internal consistency and good test-retest reliability [6]. The Swedish DERS has adequate psychometric properties [46]. In this sample, internal consistency was excellent for Impulse/Goals (α = .927), and good for the other dimensions (αs = .869 to .895).

*The SASB* intrex version [50] Introject measures self-directed behaviors using 36 items scored 0–100 (10-point increments) providing eight clusters (subscales): 1. Self-emancipation; 2. Self-affirmation; 3. Self-love; 4. Self-protection; 5. Self-control; 6. Self-blame; 7. Self-attack; and 8. Self-neglect (see Fig. 1). Six clusters (2-4 and 6-8) form the Affiliation score ranging from −100 – 100; lower scores indicate more self-attack, -blame and -neglect and higher scores indicate more self-love, -acceptance and -protection. The Affiliation score was used in replication analyses while cluster scores were used in extension analyses. The American version of SASB intrex has shown good psychometric properties [50]. The Swedish SASB intrex has shown good internal consistency (Cronbach’s α = .87, Armelius, unpublished manuscript, 2001). In this sample, internal consistency was good for Clusters 3 and 7 (αs = .845 and .830), acceptable for Clusters 2, 4, 6 and 8 (αs = .717–.789), questionable for Cluster 5 (α = .644), and poor for Cluster 1 (α = .594) which was therefore dropped from further analyses.

*The EDE-Q* [51] version 4.0 measures self-rated ED pathology in the last 28 days, with 36 items scored 0–6 providing four subscales where higher scores indicate more severe pathology, and one mean Global Score, used as outcome in mediation analyses. The EDE-Q also measures ED related behaviors. We used item 18 (frequency of OBE) to categorize AN-BP and OSFED patients (no such episode = non-OBE EDs; one or more = OBE EDs). The original EDE-Q has shown good psychometric properties [52] as has The Swedish EDE-Q [53]. In this sample, internal consistency was excellent for EDE-Q Global Score (α = .927).

### Statistical analysis

Prior to analyses (using SPSS version 24 for Mac), Mahalanobis’ distance was used to examine potential multivariate outliers in each ED group separately. No outliers were observed for the simple mediation model variables (model description below), whereas for the parallel mediation model variables, some outliers were observed (a maximum of 2, i.e., < 1%) in each group and excluded from the respective analysis. All variables were z-standardized by group, making path coefficients interpretable using effect size conventions for Pearson coefficients (small ≥ .1; medium ≥ .3; large ≥ .5). PROCESS macro version 2.16.3, Model 4 [54] was used for all mediation analyses. Statistical inference for indirect effects (mediation) was conducted by bias-corrected bootstrap confidence intervals (CI) based on 10,000 bootstrap samples. All mediation analyses were adjusted for age and BMI. To avoid Type-I error due to multiple testing, we set alpha for main analyses to *p* < .001 and used 99% bootstrap CI:s for indirect effects.

#### Simple mediation models

**Fig. 2:**
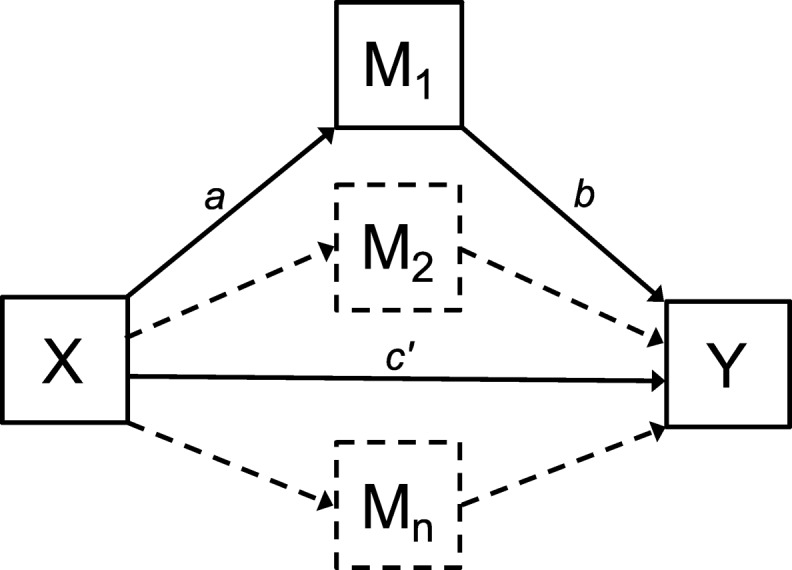
Conceptual model of mediation analysis; indirect effect of X on Y through mediator M. Components *a*, *b* and *c´* are regression coefficients. Dashed lines indicate an extended parallel mediation model; specific indirect effects through more than one mediator

The effect of independent variable X (DERS Total) on dependent variable Y (EDE-Q Global), mediated by mediator M (SASB Affiliation) was examined in both ED groups separately (see Fig. 2 for depiction of mediation model components). The opposite direction (i.e., X = SASB; M = DERS) was also examined to investigate which model best fit the data, similar to Monell et al. [9]. Simple mediation analysis yields regression coefficients for each model path: first X on M (path *a*), M on Y adjusted for X (path *b*), X on Y (total effect; path *c*), X on Y adjusted for M (direct effect; path *c´*), and lastly X through M on Y which is the product of path *a* and *b* (indirect effect; path *ab*). The direct and indirect effects are of greatest importance in this type of analysis; the significance of path *a* and *b* independently are of minor importance while their signs (positive vs. negative association) tells the direction of the indirect effect.

#### Extended parallel mediation models

Four parallel mediation models (X = 4 DERS dimensions, Y = EDE-Q Global, mediated by mediators M_1–7_ = SASB Clusters 2–8; Cluster 1 excluded due to poor reliability) were examined in both ED groups separately, regarding the total effect, direct effect (adjusted for all Ms), and seven specific indirect effects (all specific indirect effects adjusted for the others).

## Results

After describing sample characteristics, simple mediation results will be presented (one hypothesized and one alternate in each group), followed by the extended mediation models (four in each group); results in participants without OBE are reported prior to results in participants with OBE.

### Sample characteristics

Non-OBE EDs (*n* = 439) consisted of AN-R participants (*n* = 172), and AN-BP and OSFED participants lacking OBE (*n* = 35 and 232, respectively). OBE EDs (*n* = 560) consisted of BN (*n* = 350) and BED participants (*n* = 40), as well as AN-BP and OSFED participants with OBE (*n* = 29 and 141, respectively). Descriptive data on background variables and main measures and group comparisons are presented in Table 1. Since these comparisons were intended to describe and compare the subsamples, differences at *p* < .01 are reported. Participants without OBE were significantly younger, had shorter ED duration (small effects), and lower BMI than those with OBE (large effect). For the main measures, participants without OBE reported lower EDE-Q (medium effect), higher self-affirmation, lower self-neglect, higher overall Affiliation, less difficulties in goal-directed behavior and impulse control when upset, less emotional helplessness when upset, and lower overall emotion dysregulation (small effects) than participants with OBE.

**Table 1:** Descriptives for EDs without OBE (*n* = 439) and EDs with OBE (*n* = 560) on background variables and all main measures, and independent *t*-test comparisons. Cohen’s *d* (small ≥ .02, medium ≥ .05, large ≥ .08) computed for *p* < .01 differences

Variable	Non-OBE EDs		OBE EDs		***t***-value	***p***	Cohen’s ***d***
	***M (SD)***	***Min - Max***	***M (SD)***	***Min - Max***			
Age	23.46 (7.90)	16–66	25.83 (8.61)	16–72	− 4.487	<.001	.287
ED-duration	7.49 (8.12)	0–44	10.04 (8.70)	0–56	−4.739	<.001	.303
BMI	18.78 (3.91)	12.7–43.0	24.29 (6.24)	12.3–53.9	− 16.185	<.001	1.059
EDE-Q Global Score	3.50 (1.35)	0–5.8	4.15 (0.99)	0.2–6	− 8.713	<.001	.545
*DERS dimensions:*							
1. Awareness/Clarity	32.43 (8.80)	11–55	32.63 (8.68)	11–55	−.355	.723	
2. Non-Acceptance	15.91 (6.16)	6–30	16.61 (6.25)	6–30	−1.747	.081	
3. Goals/Impulse	29.64 (10.47)	11–55	32.28 (10.42)	11–55	− 3.972	<.001	.253
4. Strategies	20.33 (7.81)	8–40	22.15 (7.57)	8–40	−3.715	<.001	.236
DERS Total	98.31 (26.80)	38–176	103.66 (26.07)	38–169	−3.180	.002	.202
*SASB clusters:*							
1. Self-emancipation	30.36 (17.35)	0–82	32.28 (16.23)	0–90	−1.803	.072	
2. Self-affirmation	32.15 (21.85)	0–100	27.77 (18.17)	0–100	3.454	.001	.218
3. Self-love	32.18 (21.20)	0–100	29.00 (18.23)	0–88	2.542	.011	
4. Self-protection	42.41 (21.04)	0–100	39.71 (19.15)	0–95	2.114	.035	
5. Self-control	59.77 (17.55)	0–100	55.44 (18.06)	0–100	3.814	<.001	.243
6. Self-blame	56.48 (24.64)	0–100	59.20 (22.70)	0–100	−1.808	.071	
7. Self-attack	41.01 (26.02)	0–100	44.71 (23.80)	0–100	−2.342	.019	
8. Self-neglect	36.59 (22.46)	0–100	42.33 (21.57)	0–98	−4.094	<.001	.261
SASB Affiliation	−9.06 (39.37)	−92.5 – 99.2	−16.41 (34.01)	−89.5 – 84.3	3.162	.002	.200

*BMI* body mass index, *DERS* Difficulties in Emotion Regulation Scale, *ED* eating disorder, *EDE-Q* Eating Disorder Examination Questionnaire, *OBE* objective binge-eating episode, *SASB* Structural Analysis of Social Behavior

### Simple mediation models

DERS Total had an indirect effect on EDE-Q Global through SASB Affiliation in both groups (Table 2), such that higher levels of emotion dysregulation were associated with more negative self-directed behaviors, which in turn was associated with higher ED psychopathology. DERS had no direct effect on EDE-Q when SASB Affiliation was accounted for (sometimes referred to as “full mediation”). Descriptively, associations were slightly stronger in non-OBE than in OBE EDs. There was no evidence of the alternate indirect effect in either ED group; SASB Affiliation had significant strong direct effects on EDE-Q Global in both samples, whereas indirect effects through DERS were negligible and CI:s included zero (see Table 2).

**Table 2:** Simple mediation models examining the hypothesized model and an alternative model in EDs without OBE (*n* = 439) and EDs with OBE (*n* = 560), using standardized variables and 99% CI:s using 10,000 bootstrap samples. All models adjusted for age and BMI

Group	Path ***a***	Path ***b***	Total effect (***c***)	Indirect effect (***ab***)	99% CI	Direct effect (***c***´)	Model ***R***^**2**^
*Hypothesized direction (DERS Total - > SASB Affiliation - > EDE-Q Global)*							
Non-OBE EDs	−.718^***^	−.631^***^	.451^***^	.453	.351–.564	−.002 (n.s.)	.414^***^
OBE EDs	−.654^***^	−.479^***^	.417^***^	.313	.221–.419	.104^*^	.304^***^
*Alternative direction (SASB Affiliation - > DERS Total - > EDE-Q Global)*							
Non-OBE EDs	−.709^***^	−.002 (n.s.)	−.630^***^	.002	−.089–.095	−.631^***^	.414^***^
OBE EDs	−.652^***^	.104^*^	−.547^***^	−.068	−.152–.013	−.479^***^	.304^***^

*BMI* body mass index, *DERS* Difficulties in Emotion Regulation Scale, *CI* confidence interval, *ED* eating disorder; *EDE-Q* Eating Disorder Examination Questionnaire, *OBE* objective binge-eating episode, *SASB* Structural Analysis of Social Behavior

^*^p < .05; ^***^p < .001

### Extended parallel mediation models in EDs without OBE

**Fig. 3:**
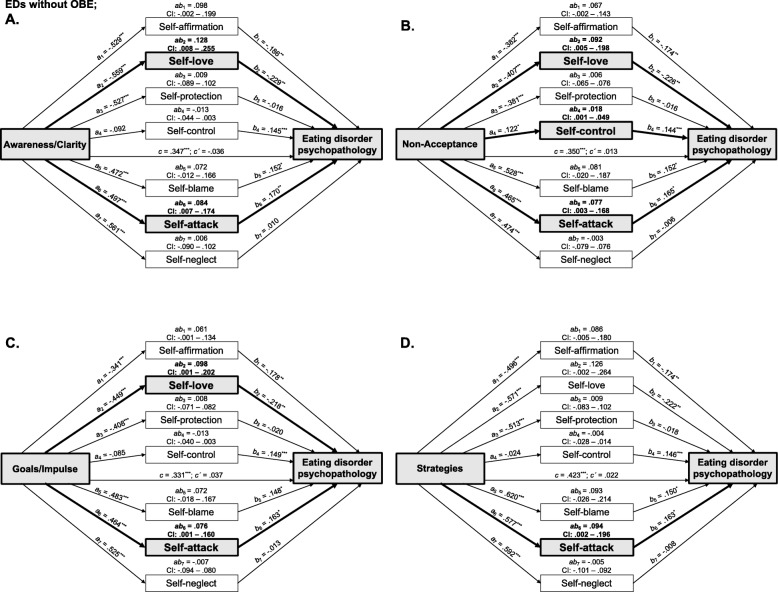
Parallel mediation models in EDs without objective binge-eating episodes positing emotion dysregulation dimensions as independent variables (**a**. Awareness/Clarity; **b**. Non-Acceptance; **c**. Goals/Impulse; **d**. Strategies), self-directed behaviors as mediators, and ED psychopathology as dependent variable. All effects are adjusted for age and BMI. Bold style and grey boxes indicate indirect effects different from zero. Path coefficients are based on standardized variables; examination of indirect effects is based 99% confidence intervals using 10,000 bootstrap samples. ^*^ = *p* < .05; ^**^ = *p* < .01; ^***^ = *p* < .001

All models were significant (*p*s < .001; *R*^2^s = .46) where DERS dimensions and the majority of SASB clusters were moderately to strongly and significantly associated (*a*-paths, see all paths for all models in Fig. 3), whereas there were only a few small significant associations between SASB clusters and ED psychopathology when controlling for DERS-dimensions (*b*-paths). In all models, each emotion dysregulation dimension first had significant moderate associations with ED psychopathology (*c-*paths; total effects), but when controlling for self-directed behaviors, these associations were non-significant (*c´-*paths; direct effects); they were instead mediated by different SASB clusters.

*Awareness/Clarity* had an indirect effect on EDE-Q such that higher difficulties in emotional awareness and clarity through lower levels of self-love and higher self-attack was associated with higher ED psychopathology. No other indirect effects had CI’s entirely above zero. *Non-acceptance* had an indirect effect on EDE-Q where higher emotional non-acceptance through lower self-love, higher self-control, and higher self-attack was associated with higher ED psychopathology. No other indirect effects had CI’s entirely above zero. *Goals/Impulse* had an indirect effect on EDE-Q where higher difficulties maintaining impulse control and goal-directed behaviors when in distress through lower self-love and higher self-attack was associated with higher ED psychopathology. *Strategies* had an indirect effect on EDE-Q where higher emotional helplessness through higher self-attack was associated with higher ED psychopathology. No other indirect effects had CI’s entirely above zero.

### Extended parallel mediation models in EDs with OBE

**Fig. 4:**
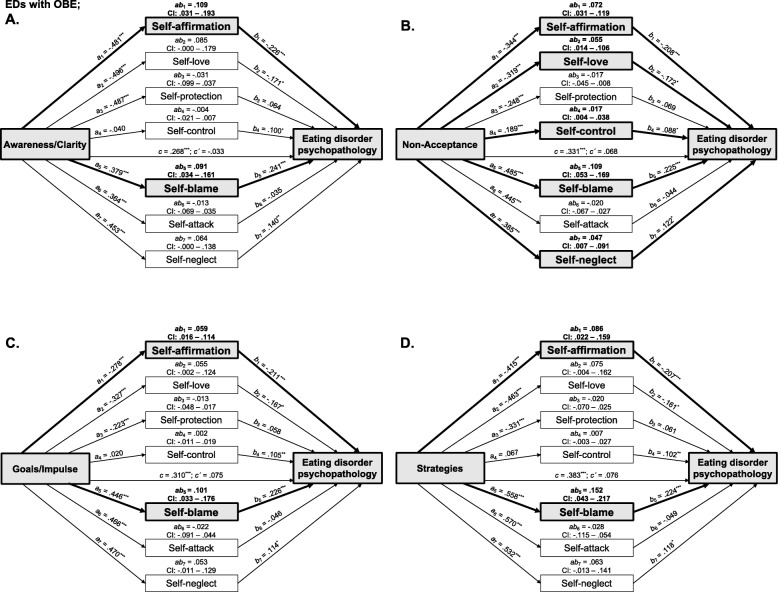
Pabrallel mediation models in EDs with objective binge-eating episodes positing emotion dysregulation dimensions as independent variables (**a**: Awareness/Clarity; **b**. Non-Acceptance; **c**. Goals/Impulse; **d**. Strategies), self-directed behaviors as mediators, and ED psychopathology as dependent variable. All effects are adjusted for age and BMI. Bold style and grey boxes indicate indirect effects different from zero. Path coefficients are based on standardized variables, examination of indirect effects is based 99% confidence intervals using 10,000 bootstrap samples. ^*^ = *p* < .05; ^**^ = *p* < .01; ^***^ = *p* < .001

Again, all models were significant (*p*s < .001; *R*^2^s = .34) where DERS dimensions and the majority of SASB clusters had significant moderate to strong associations (*a*-paths, see all paths for all models in Fig. 4), with only a few small significant associations between SASB clusters and ED psychopathology when controlling for DERS-dimensions (*b*-paths). The DERS-dimensions had small to moderate significant associations with ED psychopathology (*c-*paths), which were reduced when controlling for self-directed behaviors (*c´-*paths; direct effects), i.e., these effects were mediated by SASB clusters, albeit different ones compared to non-OBE EDs.

*Awareness/Clarity* had an indirect effect on EDE-Q such that higher difficulties in emotional awareness and clarity through lower self-affirmation and higher self-blame was associated with higher ED psychopathology. No other indirect effects had CI’s entirely above zero. *Non-acceptance* had an indirect effect on EDE-Q where higher emotional non-acceptance through lower self-affirmation, lower self-love, higher self-control, higher self-blame, and higher self-neglect was associated with higher ED psychopathology. No other indirect effects had CI’s entirely above zero. *Goals/Impulse* had specific indirect effects on EDE-Q where higher difficulties maintaining impulse control and goal-directed behaviors when in distress through lower self-affirmation and higher self-blame was associated with higher ED psychopathology. No other specific indirect effects had CI’s entirely above zero. *Strategies* had an indirect effect on EDE-Q where higher emotional helplessness through lower self-affirmation and higher self-blame was associated with higher ED psychopathology. No other indirect effects had CI’s entirely above zero.

## Discussion

The present study examined associations between trait-level emotion dysregulation, self-directed behaviors, and ED psychopathology in patients who were grouped according to absence or presence of OBE. We found that emotion dysregulation was associated with ED psychopathology through pathology specific self-directed behaviors. The simple mediation results will be summarized and discussed first, followed by the extended mediation results. Thereafter, the theoretical and clinical implications of these results will be discussed.

As hypothesized, our previous mediation finding in a sample of female students [8] was replicated in ED patients. Emotion dysregulation was strongly associated with negative self-directed behavior in both ED groups, and independently, both concepts were moderately (emotion dysregulation) and strongly (self-directed behaviors) associated with ED psychopathology, in line with prior findings [32, 55]. However, emotion dysregulation only had an indirect effect on ED psychopathology through self-directed behaviors, while self-directed behaviors had a strong association with ED psychopathology independent of emotion dysregulation. Associations were generally slightly stronger in the present sample compared to the students [8], and slightly stronger in participants without OBE. Of note, these participants scored somewhat lower ED psychopathology, overall emotion dysregulation, and negative self-directed behaviors although scoring higher self-control, the latter in line with previous findings [26]. Regarding emotion dysregulation, binge-eating participants specifically scored slightly more emotional helplessness, and difficulties focusing and maintaining control when upset, the latter in line with previous research [18].

Among participants without OBE, the most prominent mediators concerned affiliation (i.e., decreased active self-love and increased self-attack). In participants with OBE, both affiliation and autonomy were involved (i.e., decreased self-affirmation and increased self-blame). Self-blame, -love, and -affirmation have previously shown strong associations with ED psychopathology [4], but their function as potential mechanisms between emotion dysregulation dimensions and ED psychopathology are novel findings.

Although mediators differed between groups based on symptomatology, they diverged surprisingly little between models within each group. Prior work on the DERS suggests that Awareness tends to diverge, for instance by showing weaker relations with other psychopathology and the other subscales except with Clarity [46, 49]. Even so, mediators in this model were similar to the other models within each group; different emotion dysregulation dimensions seem associated with ED psychopathology through similar self-directed behaviors within each ED pathology type. Only the Non-acceptance models included additional mediators: higher self-control among participants without OBE; lower self-love, higher self-control, and higher self-neglect among participants with OBE. However, these indirect effects were smaller than others, and although SASB self-control has showed clinically relevant associations in EDs [28, 29], our self-control results needs to be interpreted with caution due to questionable scale reliability.

Possibly, other ways of grouping patients, for instance by directly assessed impulsivity, compulsivity, or overall psychiatric symptom load; or by narrowing the groups even further (e.g., contrasting highly restrictive patients with multi-impulsive ones), might have generated higher specificity, as well as clarifying the clinical relevance of the differences we saw.

### Theoretical and clinical implications

Our overall results suggest that emotion dysregulation only has an indirect effect on ED psychopathology through self-directed behaviors, whereas self-directed behaviors have a substantial direct effect on ED psychopathology. Therefore, self-directed behaviors should likely always be addressed in treatment, both independently and as an important process whereby patients may currently attempt to manage their emotions. Patterns of emotion (dys) regulation and self-directed behaviors develop in childhood within attachment relationships where the child’s emerging sense of self as capable or deficient, and worthy of encouragement and care, or of criticism and neglect, is formed in ongoing interactions. These interactions with significant others are *introjected* and become future self-directed behaviors [7], also forming the foundation of emotion regulation abilities [56, 57].

The specific indirect effects indicate differing processes and needs depending on ED presentation. Among participants without loss of control binge-eating, emotion dysregulation was associated with *lower self-love* and *higher self-attack*, which in turn was associated with greater ED psychopathology. Experiencing emotions as unacceptable was additionally associated with somewhat *higher self-control* in relation to pathology. Self-love reflects secure attachment and the experience that someone important provides safety, love, and understanding when needed. Such an experience may be particularly important when emotions are undifferentiated, uncomfortable, or feel out of control. Our results suggest that in such situations, patients who do not lose control over eating may tend to believe that they will not be met with loving care. For these patients, ED psychopathology might serve to cut them off from themselves and others, thereby suppressing the hope for love, but preserving a coherent albeit pathological sense of self. In interpersonal theory, self-hate is understood as introjected interactions characterized by attacking and recoiling, taking the form of aggressive efforts to either master, or gain distance from, perceived threats [7]. Speculatively, in these patients, emotions could represent such threats, evoking aggressive attack towards the body by engagement in ED pathology, thereby concretizing self-attack.

This perspective adds to existing models of primarily restrictive patients, such as the functional emotional avoidance model [16] and the cognitive-interpersonal maintenance model [33], both of which highlight the avoidant/alexithymic emotional processing style in restrictive patients. Our results indicate that therapists who treat restrictive EDs with cognitive-behavioral therapy (CBT) may need to help patients understand how problems regulating emotions could be translated into self-attack and lack of self-love. Interventions based on self-compassion [35] may benefit these patients if delivered with an explicit focus bridging the gap between feelings and symptoms. Psychodynamic and interpersonal therapists who may already be attending to attachment issues, may nevertheless need to place increased focus on patients’ sense of security when exploring emotional awareness and instances of self-attack, especially in the context of the therapeutic relationship.

Participants who had loss of control binge-eating displayed a more complex pattern. All emotion dysregulation dimensions were associated with *lower self-affirmation,* which in turn was associated with greater ED psychopathology. Since emotion dysregulation thereby may entail concomitant perceptions of not being met with openness and acceptance, distress tolerance and the possibility of disentangling emotional interpersonal situations may become reduced, thereby increasing the risk for patients to resort to symptoms as a form of distraction. All emotion dysregulation dimensions were also associated with *higher self-blame*; difficulties in emotional acceptance additionally implied *higher self-neglect*, *higher self-control* and *lower self-love*, in turn associated with greater ED psychopathology. While both self-blame and self-neglect are hostile, self-blame also encompasses aspects of control whereas self-neglect implies relinquishing control. In interpersonal theory, self-blame parallels interpersonal interactions characterized by blaming and appeasing; self-neglect parallels ignoring and walling off. Trying to appease a hostile and controlling other (internally or externally perceived) may necessitate hypervigilance until whatever is perceived as threatening has receded, either by aggressive actions or depressive withdrawal [7]. In the context of threatening emotions, these patients may anxiously oscillate between hostile self-control or by giving up. The former might spur increased efforts of perfectionistic restraint, whereas the latter might spur further loss-of-control symptoms (cf. the “what-the-hell effect” where minor violations of a perfectionistic dietary rule leads to overindulgence) [58]. However, as we only modelled global ED psychopathology, such differentiated outcomes are only speculations.

These findings contribute to dysregulation models of binge-eating pathology [5, 21, 34] by suggesting inclusion of self-blame and spontaneity (in both positive and negative ways) as key processes to highlight in treatment, in that they may represent subjectively valid links between emotions and symptoms. In CBT, learning to tolerate anxiety, disentangling distressing events, and increasing curiosity towards one’s mental states, while neither trying to increase negative control nor giving up, might be particularly important. Psychodynamic and interpersonal therapists could explore associations between emotions, negative self-control, and symptoms, while helping patients to understand how relationships inside and outside therapy impact on symptoms.

Our results also suggest that therapists need to be mindful of their own reactions, regardless of psychotherapeutic approach, in order not to maintain problematic regulation processes [7]. For instance, a patient who walls off and increases ED symptoms might trigger expressions of frustration or anger in the therapist, which in turn risks confirming the patient’s pathological self-related beliefs and emotion regulation strategies. If therapists instead show interest, acceptance, and validation, while openly acknowledging and discussing challenges to the therapeutic process, patients may find it easier to move from self-hostility toward self-affiliation.

### Strengths and limitations

This study had several strengths. Our large, nationally representative clinical sample, including most DSM-5 EDs, strengthened the ecological validity of findings. Additionally, the study concurrently examined both emotional and self-related factors previously shown to be meaningful in EDs, and the analysis of these factors in two clinically relevant sub-samples allowed high specificity. There are, however, also several important limitations.

First, since we only had trait-level cross-sectional data, we cannot infer causality. Although statistical mediation, including contrasting with models stipulating the opposite direction of effect, might indicate the possibility of a causal association, other research designs are needed to support such claims. Second, we had no control over DERS administration, possibly biasing the sample. Although there were no differences in patient related variables (e.g., ED pathology, self-directed behaviors, depression, anxiety, age) depending on if patients were administered the DERS or not, there might be unknown factors that affect the generalizability of the results. Third, the results may only generalize to treatment-seeking females. Fourth, although the DERS variables used in this study are based on Gratz and Roemer’s original and clinically relevant conceptualization of emotion dysregulation [6], they are not psychometrically examined and validated in clinical samples. A lower-order correlated traits model including these four as the traits has showed bad fit in university students [59], but no study to our knowledge has examined this solution or alternative second-order or bifactor four-factor solutions in clinical samples (where models showing good fit in control samples often do not fit as well) [46, 49]. However, subscale intercorrelations (Tables S1 and S2), theoretical groundwork [6, 13, 60], and prior findings regarding unique contribution of individual subscales [46, 49, 61] indicate that the combination of Awareness/Clarity and Goals/Impulse is theoretically relevant and empirically reasonable. Fifth, the patient categorization in this study is not commonly accepted, and we only considered OBE but not purging when dividing participants. However, some prior findings point to relevant differences depending on presence/absence of binge-eating in EDs [39, 62], and stronger unique associations between binge-eating and negative urgency compared to those with purging has been reported in female students [40]. Additionally, there were some relevant differences, and results did differ, between samples which provides some validity to this categorization when considering the constructs under consideration here. Even so, future research needs to examine the potential impact of both binge-eating and purging further. Sixth, in the extended mediation analysis, the use of 99% CI when boot-strapping indirect effects undoubtedly widened the CI:s which, in combination with the inclusion of seven more or less correlated mediators in each model, likely contributed to the observed indirect effects being relatively few and rather weak [54]. Our rationale was due to the exploratory nature of this study and lack of previous findings, although our large samples likely counteracted some uncertainty in the results. Future replication could select the most prominent mediators to refine the mediation models. Lastly, the internal consistency was poor for SASB Cluster 1 and questionable for Cluster 5. We chose to exclude Cluster 1, while all results including Cluster 5 needs to be interpreted with great caution. Internal consistency was acceptable to excellent for all other variables.

## Conclusions

We found ED pathology specific association pathways whereby emotion dysregulation was associated with more negative self-directed behaviors, which in turn were associated with greater ED psychopathology. This indicates that addressing patients’ self-directed behaviors could be an important way of helping patients deal with their emotions in relation to their ED psychopathology. In participants without loss of control over eating, lower self-love and higher self-attack were influential factors, whereas in participants with loss of control over eating, lower self-affirmation and higher self-blame were influential.

## Supplementary information

**Additional file 1: Table S1.** Intercorrelations between DERS scales, SASB Affiliation Score, EDE-Q Global score, age, ED duration and BMI in participants without objective binge-eating episodes. *N* = 439.

**Additional file 2: Table S2**. Intercorrelations between DERS scales, SASB Affiliation Score, EDE-Q Global score, age, ED duration and BMI in participants with objective binge-eating episodes. *N* = 560.

## Data Availability

Data belong to the Stepwise registry and are not available for sharing.

## Abbreviations

AN: Anorexia nervosa
AN-R: Anorexia nervosa restricting subtype
AN-BP: Anorexia nervosa binge/purge subtype
BED: Binge eating disorder
BN: Bulimia nervosa
CBT: Cognitive behavioral therapy
CI: Confidence interval
DERS: Difficulties in Emotion Regulation Scale
DSM: Diagnostic and Statistical Manual of mental disorders
ED: Eating disorder
EDE-Q: Eating Disorder Examination Questionnaire
OBE: Objective binge-eating episode
OSFED: Other specified feeding and eating disorders
SASB: Structural Analysis of Social Behavior
SEDI: Structured Eating Disorder
UFED: Unspecified feeding and eating disorders
