# Supplementary material for: Self-directed behaviors differentially explain associations between emotion dysregulation and eating disorder psychopathology in patients with or without objective binge-eating
Source: J Eat Disord. 2020 May 1;8:17. doi: 10.1186/s40337-020-00294-4 (PMC7193412; doi:10.1186/s40337-020-00294-4)
Supplement: Supplementary file 1 — Additional file 1: Table S1. Intercorrelations between DERS scales, SASB Affiliation Score, EDE-Q Global score, age, ED duration and BMI in participants without objective binge-eating episodes. N = 439. [file 40337_2020_294_MOESM1_ESM.docx]

Supplementary information

| **Table S1.** Intercorrelations between DERS scales, SASB Affiliation Score, EDE-Q Global score, age, ED duration and BMI in participants without objective binge-eating episodes. *N*=439. | | | | | | | | | | | | |
| --- | --- | --- | --- | --- | --- | --- | --- | --- | --- | --- | --- | --- |
| **Variables** | **1** | **2** | **3** | **4** | **5** | **6** | **7** | **8** | **9** | **10** | **11** | **12** |
| 1. Non-Acceptance | – |  |  |  |  |  |  |  |  |  |  |  |
| 2. Goals | .392^***^ | – |  |  |  |  |  |  |  |  |  |  |
| 3. Impulse | .409^***^ | .637^***^ | – |  |  |  |  |  |  |  |  |  |
| 4. Awareness | .314^***^ | .213^***^ | .282^***^ | – |  |  |  |  |  |  |  |  |
| 5. Strategies | .568^***^ | .708^***^ | .697^***^ | .313^***^ | – |  |  |  |  |  |  |  |
| 6. Clarity | .441^***^ | .466^***^ | .485^***^ | .541^***^ | .513^***^ | – |  |  |  |  |  |  |
| 7. Total score | .709^***^ | .772^***^ | .796^***^ | .562^***^ | .878^***^ | .742^***^ | – |  |  |  |  |  |
| 8. SASB Affiliation | -.514^***^ | -.409^***^ | -.510^***^ | -.551^***^ | -.647^***^ | -.502^***^ | -.703^***^ | – |  |  |  |  |
| 9. EDE-Q Global | .348^***^ | .237^***^ | .314^***^ | .293^***^ | .412^***^ | .267^***^ | .425^***^ | -.625^***^ | – |  |  |  |
| 10. Age | -.030 | -.101^*^ | -.130^**^ | -.017 | -.101^*^ | -.127^**^ | -.113^*^ | -.054 | .094^*^ | – |  |  |
| 11. ED duration | .042 | -.003 | -.029 | .061 | .003 | -.066 | .003 | -.128^**^ | .132^**^ | .815^***^ | – |  |
| 12. BMI | -.020 | -.104^*^ | -.064 | -.051 | -.021 | -.192^**^ | -.091 | .049 | .118^*^ | .151^**^ | .169^**^ | – |
| Note: ^*^ *p*<.05; ^**^ *p*<.01; ^***^ *p*<.001. BMI = body mass index; DERS = Difficulties in Emotion Regulation Scale; ED = eating disorder; EDE-Q = Eating Disorder Examination Questionnaire; SASB = Structural Analysis of Social Behavior. | | | | | | | | | | | | |
