# Supplementary material for: Self-directed behaviors differentially explain associations between emotion dysregulation and eating disorder psychopathology in patients with or without objective binge-eating
Source: J Eat Disord. 2020 May 1;8:17. doi: 10.1186/s40337-020-00294-4 (PMC7193412; doi:10.1186/s40337-020-00294-4)
Supplement: Supplementary file 2 — Additional file 2: Table S2. Intercorrelations between DERS scales, SASB Affiliation Score, EDE-Q Global score, age, ED duration and BMI in participants with objective binge-eating episodes. N = 560. [file 40337_2020_294_MOESM2_ESM.docx]

Supplementary information

| **Table S2.** Intercorrelations between DERS scales, SASB Affiliation Score, EDE-Q Global score, age, ED duration and BMI in participants with objective binge-eating episodes. *N*=560. | | | | | | | | | | | | |
| --- | --- | --- | --- | --- | --- | --- | --- | --- | --- | --- | --- | --- |
| **Variables** | **1** | **2** | **3** | **4** | **5** | **6** | **7** | **8** | **9** | **10** | **11** | **12** |
| 1. Non-Acceptance | – |  |  |  |  |  |  |  |  |  |  |  |
| 2. Goals | .459^***^ | – |  |  |  |  |  |  |  |  |  |  |
| 3. Impulse | .512^***^ | .658^****^ | – |  |  |  |  |  |  |  |  |  |
| 4. Awareness | .173^***^ | .114^**^ | .207^***^ | – |  |  |  |  |  |  |  |  |
| 5. Strategies | .622^***^ | .696^***^ | .721^***^ | .263^***^ | – |  |  |  |  |  |  |  |
| 6. Clarity | .403^***^ | .370^***^ | .388^***^ | .556^***^ | .473^***^ | – |  |  |  |  |  |  |
| 7. Total score | .740^***^ | .758^***^ | .812^***^ | .489^***^ | .887^***^ | .688^***^ | – |  |  |  |  |  |
| 8. SASB Affiliation | -.462^***^ | -.395^***^ | -.443^***^ | -.469^***^ | -.594^***^ | -.450^***^ | -.642^***^ | – |  |  |  |  |
| 9. EDE-Q Global | .336^***^ | .314^***^ | .272^***^ | .200^***^ | .394^***^ | .299^***^ | .416^***^ | -.543^***^ | – |  |  |  |
| 10. Age | -.017 | -.084^*^ | -.093^*^ | -.044 | -.109^*^ | -.125^**^ | -.106^*^ | -.059 | -.027 | – |  |  |
| 11. ED duration | .034 | -.033 | -.043 | -.007 | -.032 | -.083 | -.034 | -.106^*^ | .003 | .837^***^ | – |  |
| 12. BMI | .057 | -.015 | -.002 | -.018 | .010 | -.094^*^ | -.007 | -.093^*^ | .033 | .302^***^ | .321^***^ | – |
| Note: ^*^ *p*<.05; ^**^ *p*<.01; ^***^ *p*<.001. BMI = body mass index; DERS = Difficulties in Emotion Regulation Scale; ED = eating disorder; EDE-Q = Eating Disorder Examination Questionnaire; SASB = Structural Analysis of Social Behavior. | | | | | | | | | | | | |
